# Association between cognitive performance and sarcopenic obesity in older adults with Alzheimer’s disease

**DOI:** 10.1590/1980-5764-DN-2021-0039

**Published:** 2022

**Authors:** Timothy Gustavo Cavazzotto, Caroline do Valle de Campos, Caryna Eurich Mazur, Danilo Fernandes da Silva, Juliana Maria Silva Valério, Edgar Ramos Vieira, Weber Claudio Francisco Nunes da Silva, Juliana Sartori Bonini

**Affiliations:** 1 Universidade Estadual do Centro-Oeste, Departamento de Educação Física, Guarapuava PR, Brazil.; 2 Universidade Estadual do Centro-Oeste, Departamento de Nutrição, Guarapuava PR, Brazil.; 3 Faculty of Health Sciences, School of Human Kinetics, Ottawa, Ontario, Canada.; 4 Instituto Federal do Paraná, Palmas PR, Brazil.; 5 Florida International University, Department of Physical Therapy, Miami, Florida, USA.; 6 Universidade Estadual do Centro-Oeste, Laboratório de Neuropsicofarmacologia, Guarapuava PR, Brazil.; 7 Universidade Estadual do Centro-Oeste, Laboratório de Neurociência e Comportamento, Guarapuava PR, Brazil.

**Keywords:** Body Composition, Sex Characteristics, Dementia, Composição Corporal, Caracteres Sexuais, Demência

## Abstract

Sarcopenic obesity (SO), the co-occurrence of sarcopenia and obesity, is associated with functional loss, frailty, and incapacity in older adults. Recently, SO was associated with reduced cognitive performance in adults. However, no SO studies have been done with older adults with Alzheimer’s disease (AD).

**Objective:**

The objective of this study was to verify the occurrence of SO and associated factors in 43 older adults with AD.

**Methods:**

We applied the Mini-Mental State Examination (MMSE) and Clinical Dementia Rating (CDR). SO was verified by using dual-emission X-ray absorptiometry.

**Results:**

We found five women with SO. Women had higher body fat and lower muscle mass compared with men. There was a significant relationship between body fat and cognitive performance only in men (r=0.65; p<0.01) adjusted by age and education. Men with obesity and aged >75 years had better cognitive performance compared with non-obese men aged <75 years (p=0.010) and women with obesity aged >75 years (p=0.033).

**Conclusions:**

Women with AD had higher body fat and lower muscle mass than men. SO occurs in older women with AD. Men with higher body fat showed better cognitive performance, independent of age and education.

## RESUMO.

A obesidade sarcopênica (SO), coocorrência de sarcopenia e obesidade, está associada à perda funcional, à fragilidade e à incapacidade em idosos. Recentemente, verificou-se que a SO está associada ao desempenho cognitivo reduzido em adultos. No entanto, não foram feitos estudos de SO em idosos com doença de Alzheimer (AD).

**Objetivo:**

Verificar a ocorrência de obesidade sarcopênica e fatores associados em 43 adultos idosos com doença de Alzheimer.

**Métodos:**

Aplicamos o miniexame do estado mental (MEEM) e a avaliação clínica da demência (CDR). A SO foi verificada utilizando a absorciometria de dupla emissão de raios X.

**Resultados:**

Foram classificadas cinco idosas com SO. As mulheres idosas tinham maior gordura corporal e menor massa muscular em comparação com os homens. Houve relação significativa, ajustada por idade e educação, entre gordura corporal e desempenho cognitivo apenas nos homens (r=0,65; p<0,01). Os homens com obesidade e com mais de 75 anos tiveram melhor desempenho cognitivo em comparação com os homens não obesos <75 anos (p=0,010) e com as mulheres com obesidade >75 anos (p=0,033).

**Conclusões:**

As mulheres com AD tinham maior gordura corporal e menor massa muscular do que os homens. A SO ocorreu em mulheres mais velhas com AD. Os homens com maior gordura corporal apresentaram melhor desempenho cognitivo, independentemente da idade e da educação.

## INTRODUCTION

Alzheimer’s disease (AD), the most prevalent dementia, affects 40–50 million people worldwide^1^. AD is a neurodegenerative disorder involving progressive decline of episodic memory and cognitive and functional capacities. Environmental and other factors are associated with increased risk for AD. Cerebrovascular diseases, diabetes, hypertension, obesity, and dyslipidemia are the most commonly reported health-related risk factors. Recently, sarcopenic obesity (SO) was associated with reduced cognitive performance in adults^2^. SO is the co-occurrence of sarcopenia and obesity with low lean and very high-fat mass. Sarcopenia is associated with functional loss, frailty, incapacity, and cognitive impairment^3^. Obesity is associated with inflammation and metabolic comorbidities. However, the association between obesity and cognitive impairment is controversial and influenced by the accuracy of adiposity measures. Depending on age, obesity can be either a risk or a protective factor for dementia. In adults aged <65 years, obesity increased the risk of dementia (relative risk [RR]: 1.41; 95%CI 1.20–1.66). In contrast, obesity was a protective factor for adults aged >65 years (RR: 0.83; 95%CI 0.74–0.94)^4^. In a population-based study, the SO rates were 12.6% in men and 33.5% in women aged >60 years old, and 27.5% in men and 48% in women aged >80 years^5^. SO is more prevalent in women, people with diabetes and dyslipidemia, and/or among those who are sedentary^6^. However, the prevalence of SO and risk factors among those with AD is unclear. Previous studies have evaluated obesity and sarcopenia independently from each other in older adults with AD, or the relationship between SO and cognitive decline in older adults without AD,^7^ but no studies of SO have been done with older adults with AD. Therefore, we examined the occurrence of SO and the relationship between body composition, age, and cognitive performance with SO in older adults with AD.

## METHODS

### Sample and study design

A total of 43 people (23 men, 20 women, 77±7 years old) with medically diagnosed AD based on the National Institute of Neurologic and Communicative Disorders and Stroke and the Alzheimer’s Disease-Related Disorders Association (NINCDS-ADRDA) criteria participated in this study as volunteers. Informed consent has been obtained from patients and/or caregivers. Two trained researchers accompanied by caregivers performed the anamnesis, cognitive evaluations, and anthropometric measurements at the participants’ homes. Subsequently, the participants went to a laboratory for the body composition evaluation using dual-emission X-ray absorptiometry (DEXA). The exclusion criteria were having incomplete data in the Mini-Mental State Examination (MMSE), the Clinical Dementia Rating (CDR), or the DEXA examination. A total of 18 men and 18 women had complete data sets and composed the final sample (n=36). The authors assert that all procedures contributing to this work comply with the relevant national and institutional committees’ ethical standards on human experimentation and with the Declaration of Helsinki 1975, as revised in 2008. The Ethics Committee approved the research protocol (# 3.363.878).

### Measures

#### Body Composition:

We measured body weight in kilograms (0.01 kg) and height in centimeters (0.1 cm) by using an electronic scale and fixed stadiometer, applying standardized procedures. Total fat (%), lean mass (kg), and appendicular muscle mass (kg) were obtained from the dual-emission X-ray absorptiometry (DEXA) examinations. Obesity was classified based on body fat >28% for men and >39% for women (Cooper Institute for DEXA measurement).

#### Cognitive Assessment:

We used the MMSE with scores ranging from 0 to 30, with higher values representing better cognitive performance. AD stage was classified as mild, moderate, or severe using the CDR 1, CDR 2, or CDR 3, respectively.

#### Sarcopenia:

It was assessed based on appendicular skeletal muscle mass index (ASMI) <7.2 for men and <5.7 for women. ASMI is the sum of the muscle mass of the four limbs divided by the square of the height in meters (ASMI=ASM/height^2^).

#### Sarcopenic Obesity:

It was assessed based on residual values of regression model less than -2.3 for men and less than -3.4 for women after adjusting muscle mass by fat mass and height^7^.

### Statistical analysis

We analyzed the data using *Statistical Package for the Social Sciences* (SPSS), version 25.0. The results were described as means and standard deviations or relative frequencies. Normality distribution was tested and confirmed by using the Shapiro-Wilk test. To compare age, education, and body composition between men and women, we used Student’s *t*-test. The relationship between body composition (e.g., body fat, ASMI, and residual values) and MMSE was assessed by using the Pearson correlation. The same relationships were tested, controlling by age and years of education (i.e., partial correlation). We compared the MMSE results among subgroups (e.g., 1 — non-obese and non-sarcopenic, 2 — obese, 3 — sarcopenic, and 4 — SO) using one-way analysis of variance (ANOVA) and analysis of covariance (ANCOVA), using age and years of education as covariates, with the Bonferroni post hoc tests. We clustered the older adults according to age (using the median as the cutoff point) and obesity/sarcopenic groups. We compared MMSE values using two-way ANOVA (sex × age — obesity/sarcopenic) with the Bonferroni post hoc tests, with years of education as covariates. The alpha value was set to be p<0.05.

## RESULTS

Men had significantly higher weight, ASMI, and residual value than women. Women had approximately 10% higher body fat and lower muscle mass and weight (p<0.05; Table 1).

**Table 1:** Body composition and Mini-Mental State Examination findings.

	All n=36		Men n=18		Women n=18		Sex differences	
	M	SD	M	SD	M	SD	t	p-value
Age	77.6	7.37	76.3	6.2	79.1	8.2	-1.3	0.204
Education	3.9	3.6	4.1	4.0	3.7	3.2	-0.3	0.724
Weight	66.5	10.8	70.9	7.5	61.5	12.1	3.1	0.003
MMSE	13.6	6.1	14.0	7.7	13.1	4.1	0.5	0.644
Body fat	35.9	8.9	31.1	6.6	41.2	8.4	-4.2	<0.001
ASMI	6.3	1.2	7.1	0.9	5.5	0.8	5.8	<0.001
Residual value	-0.99	3.26	1.24	2.42	-3.45	2.08	6.5	<0.001

MMSE: Mini-Mental State Examination; ASMI: appendicular muscle mass index (ASMI=ASM/height^2^); residual: regression model adjusting muscle mass by fat mass and height^7^; M: mean; SD: standard deviation.

**Figure 1(A):**
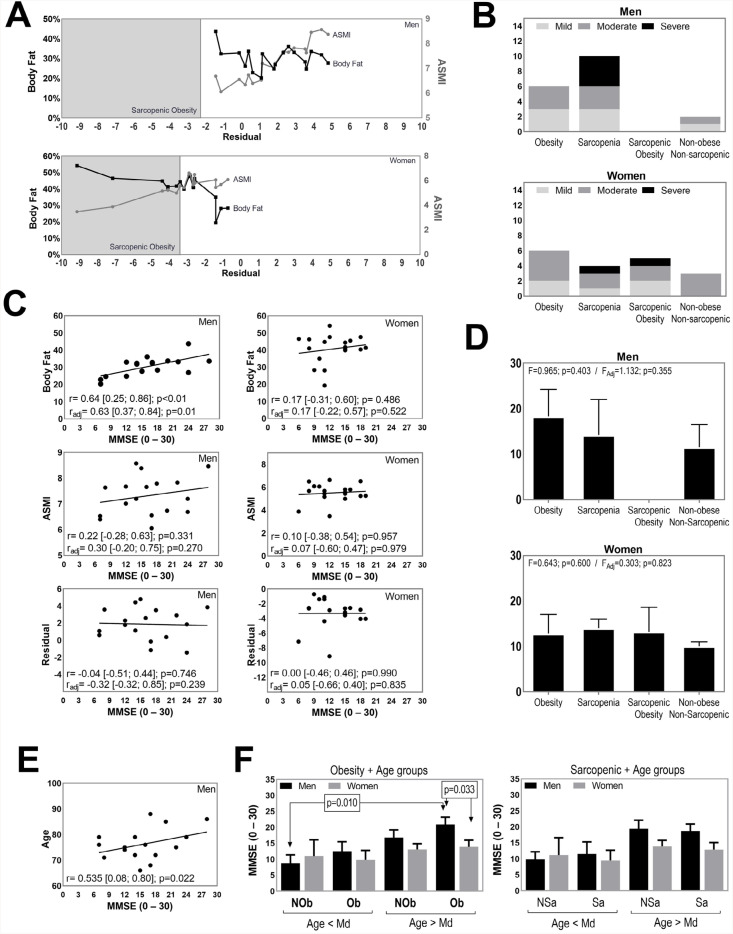
(A) Case-by-case appendicular skeletal muscle mass index, body fat, and residual values by sex. (B) Occurrence of obesity, sarcopenia, and sarcopenic obesity by Alzheimer’s disease stage. (C) Relationship between appendicular skeletal muscle mass index, body fat, and residual value with Mini-Mental State Examination, Pearson correlation (r), and partial correlation adjusted by age and education (r_adj_). (D) Sex-related differences for Mini-Mental State Examination by clinical condition, unadjusted and adjusted by age and education (F_adj_). (E) Relationship between age and Mini-Mental State Examination. (F) Mini-Mental State Examination comparison by subgroups combining age and obesity or sarcopenia.

Figure 1A presents the results for body fat, ASMI, and residual value. Five women had SO: two had mild dementia, two had moderate dementia, and one had severe dementia (Figure 1B). Two women and four men had severe AD. The four men with severe AD also had sarcopenia (Figure 1B). Only in men, MMSE and body fat were significantly correlated with each other (r=0.64; 95%CI 0.25–0.86; p<0.01]) even when adjusted by age and education (r=0.63; 95%CI 0.37–0.84; p=0.01]) (Figure 1C). Higher MMSE values were observed in obese men; however, there were no significant differences among subgroups (Figure 1D). Age was not associated with body composition. However, cognitive performance was higher for men in older age (r=0.54; p=0.022) (Figure 1E). Obese men (>75 years) had better cognitive performance compared with non-obese men (<75 years) and women with obesity (>75 years), independent of education (Figure 1F).

## DISCUSSION

We examined the occurrence and factors associated with SO in 36 older adults with AD. To the best of our knowledge, this is the first study to describe SO using DEXA measurements in older adults with AD. Few studies investigated the relationship between body composition and cognitive performance in patients with AD using indirect fat and/or muscle mass measures like circumferences, body mass index (BMI), or bioelectrical impedance. These techniques affect the diagnostic accuracy for sarcopenia and obesity^8^. The use of DEXA allowed better measurements of muscle and fat tissues in our study. In our study, six women and one man had SO based on ASMI and body fat results from the DEXA examination. Similar to a previous study^6^, our results indicate a higher prevalence of SO in women than in men. Besides, women had higher body fat and a lower appendicular muscle mass. In general, women are more susceptible to the development of osteoarticular diseases, the decline of strength and muscle mass, and obesity.

Both the observed relationship between fat and MMSE in men and the higher MMSE values in obese older men reveal the importance of new studies exploring the age-related role of body fat and cognitive ability. From the few systematic reviews and experimental studies available in the literature, the results are still controversial and dependent on the measures used^4^. Obesity in middle-aged adults increases the risk for AD and may have a protective role on the aging brain in older adults^4^. In our results, non-sarcopenic and older men showed the same cognitive performance as other age groups. In contrast, obese men aged >75 years had significantly higher MMSE values. Experimental studies are needed to explain the mechanisms involved in the age-dependent opposite effects of obesity on cognitive functions.

SO is associated with hospitalizations and lower survival time^9^. Exercises, healthy eating, and weight management interventions are recommended to prevent and treat SO^10^. However, a restrictive diet^11^ and pharmacological therapies can concomitantly decrease fat and muscle mass, increase sarcopenia, affect behavior (e.g., stimulate aggressiveness), and/or intensify the AD degenerative process. Therefore, there is an essential gap for experimental clinical studies regarding therapies to treat SO.

The limitations of our study include the lack of muscle function measures related to sarcopenia, the small sample size, and the possible survival bias since the study participants were volunteers from the community. However, the evaluations were performed using gold-standard measures and analytical recommendations, and the results provide relevant insights for future research and practice.

In conclusion, women had higher body fat and lower muscle mass than men with AD; five women had SO. Men with higher body fat showed better cognitive performance, independent of age and education.
